# Realist evaluation of an enhanced health visiting programme

**DOI:** 10.1371/journal.pone.0180569

**Published:** 2017-07-03

**Authors:** Lawrence Doi, Ruth Jepson, Samantha Hardie

**Affiliations:** 1 Scottish Collaboration for Public Health Research and Policy, Usher Institute of Population Health Sciences and Informatics, University of Edinburgh, Edinburgh, United Kingdom; 2 School of Health and Life Sciences, Glasgow Caledonian, Glasgow, United Kingdom; University of Stirling, UNITED KINGDOM

## Abstract

**Background:**

The health visitors’ role in many countries is changing. In Scotland, the role has undergone substantial changes through the introduction of an enhanced health visiting programme, which includes increased, structured home visits. This evaluation was conducted within NHS Ayrshire and Arran, one of the 14 Scottish Health Boards. Our aim was to understand and explain how, and why, the programme could contribute to improving health and wellbeing outcomes for children and families.

**Methods:**

We used a realist evaluation approach, conducted in three phases. In phase one, eight managerial staff involved in developing and implementing the programme provided data, which were used to develop initial programme theories. In phase two, the programme theories were tested using qualitative data from 25 health visitors and 22 parents. The programme theories were refined through analyses and interpretation of data in phase three.

**Results:**

The home visiting context provided by the programme interacted with the mechanisms of the programme and produced outcomes such as early identification of health and wellbeing issues amongst families who needed more support, leading to referral and engagement with sources of additional help. The home visits facilitated development of parent-health visitor relationships, and parents considered health visitors as their first point of contact on children’s wellbeing and developmental-related issues. Moreover, the programme provided more clarity to health visitors’ role, which in turn enhanced partnership working. However, there were aspects of the programme that may require further development. For instance, both parents and health visitors were concerned about the wide gaps between some home visits.

**Conclusions:**

The enhanced health visiting programme increased opportunities for monitoring and early identification of health and wellbeing concerns. It created structures for a more efficient partnership working and ensured that the needs of children and families were supported. These benefits need to be evaluated further in an effectiveness study.

## Introduction

The provision of a quality healthcare programme that promotes universal access to healthcare in early years has the potential to reduce health inequalities in later life [1, 2]. An example in the UK is the universal child health programme, often but not solely provided by the public health nursing services, and delivered by health visitors (may be called public health nurses or community nurses in other countries). Historically, health visiting has had a preventive nursing role and encompassed an integrated package of immunisation, screening, surveillance, health promotion and parenting support for families and children from birth to about 5 years of age [3, 4]. In the USA, early public health nursing roles also included advocacy, community organising, health education, and political and social reform, but this has changed to focus more on collaboration and partnerships with communities to address social problems in recent years [5]. The ultimate goal of most health visiting or public health nursing role is to improve outcomes for children, in spite of subtle differences that may exist across countries.

Recently, the health visiting service has undergone substantial changes in Scotland. These changes are in line with the national Getting It Right For Every Child (GIRFEC) agenda of improving outcomes and supporting the wellbeing of children and young people [6]. Another policy document published in 2013, Public Health Nursing Services–Future Focus: CEL 13 [7], set out the recommendation that the health visitor’s role, responsibilities and titles, as defined in Nursing for Health review [8], needed to be refocused as part of an enhanced health visiting programme.

Although several components of health visiting have been found to be effective [4], it is recognised that any programme does not work everywhere or for everyone, and that context actually makes a difference to programme outcomes [9, 10]. Indeed, because actors make particular decisions in response to a programme, the reasoning of the actors in response to the resources or opportunities provided by the programme (mechanisms) produces the outcomes [9]. Therefore in order to understand how a programme works to produce intended and unintended outcomes, it is important to interrogate the interaction between contexts and mechanisms. The aim of this evaluation was to understand and explain how, and why, the enhanced health visiting (EHV) programme could contribute to improving health and wellbeing outcomes for children and families.

### The enhanced health visiting programme

The enhanced health visiting programme in Scotland is underpinned by available research evidence, policy direction and priorities. All Scottish National Health Service (NHS) Boards are expected to implement the enhanced service in line with CEL 13. However, NHS Ayrshire and Arran, one of the NHS Boards, was the first to adopt the EHV programme in mid-2013. The EHV programme provides universal assessment pathways that ensure increased, structured home visits. Following an initial assessment, families are categorised as either ‘core’ or ‘additional’ based on the level of support needed. Core families, who do not usually require additional monitoring and support, will receive a minimum of eleven visits from their health visitors. The first visit occurs at 11–14 days after birth, then weekly until the fifth week. The next visit is at 6–8 weeks, which is followed by visits at 12, 16 and 24 weeks. The next two visits occur at 12 months and 27–30 months. The last visit is the preschool handover assessment contact. Additional information of what each assessment or review visit entails is available elsewhere [11]. Within that period, ‘additional’ programmes of care can be offered to children and families if required. This is in line with the proportionate universalism concept described by Marmot et al. [12], which is an attempt to reduce the steepness of the social gradient in health. This means that, even though EHV is universal in nature, its delivery is proportionate to the level of disadvantage.

As part of the enhanced service, there was discontinuation of some previous health visiting roles. For instance, health visitors were no longer required to either immunise children or be involved in drop-in clinics. These role changes were considered crucial because it afforded health visitors the capacity to deliver the extra elements of the enhanced programme.

## Methods

### Evaluation design

We used a realist evaluation approach, guided by RAMESES II reporting standards for realist evaluation [13]. This approach was used because a simple binary of success and failure is sometimes unhelpful when evaluating the complexities of changing services and systems [14]. Realist evaluation argues that in order for evaluation to be useful to decision makers, it needs to explain ‘what works for whom, how, in what circumstances and why’ [9, 15]. Realist evaluation focuses on building, testing and refining programme theories. This is done by exploring the complex and dynamic interaction of context (settings or conditions in which the programme was implemented), mechanisms (causal forces, powers, processes or interactions that generate change within a programme) and outcomes (intended and unintended effects) [9, 16, 17, 18]. We were keen to understand how participants, embedded within context, triggered mechanisms of the programme to produce outcomes.

**Fig 1:**
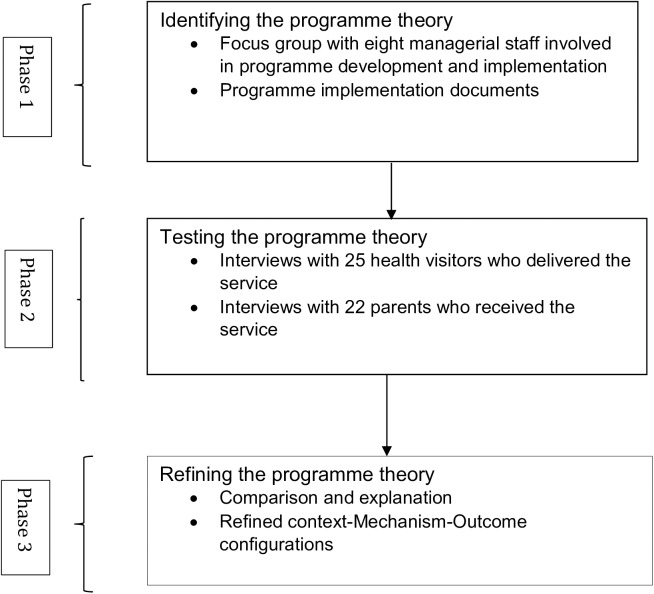
The realist evaluation process.

This evaluation unfolded in three overarching phases (Fig 1), which reflects the broad stages of realist evaluation: developing, testing and refining programme theory [19].

### Setting

NHS Ayrshire and Arran, was the first to introduce the enhanced service in mid-2013. It is one of fourteen Scottish health boards. It is located in the mid-south west of Scotland and covers an area of 750,464 square hectares. It had about 80 health visitors at the time of data collection in 2015.

### Participants

Participants for the study included key stakeholders, health visitors and parents. Key stakeholders were those involved in designing and overseeing the implementation of the enhanced service at the health board level. Health visitors were those who had received training on the enhanced service and all had experience of delivering the enhanced service. Parents included those who had received the service, with some having experience of the previous service model.

### Recruitment and data collection for phases 1 and 2

#### Phase 1. Identifying the programme theory

All programmes have implicit or explicit programme theory or theories [20, 21]. A programme theory is the assumption about how a programme is expected to achieve it desired outcomes [21]. A key principle of realist evaluation is to make the assumptions of the programme developers and implementers explicit. Engaging stakeholders to unpack these assumptions is useful in realist evaluation [22]. As such, key stakeholders involved in either designing or implementing the EHV service in NHS Ayrshire and Arran were invited to participate in a focus group. The focus group explored their accounts of the purpose and key aspects of the EHV programme; its implementation; how it was expected to work; and its anticipated outcomes on families and health visiting practice (see S1 Appendix. Topic guide for Stakeholders). LD and RJ conducted the focus group with eight key stakeholders. The focus group was audio recorded and transcribed.

We used the focus group data, complemented by programme documents (for example, a logic model designed for the proposed national EHV programme), to unearth the assumptions underpinning the EHV programme, and developed initial programme theories.

#### Phase 2. Testing and refining the programme theory

The initial programme theories (see results) were tested and refined to realist evaluation programme theories. Health visitors and parents provided interview data for testing the programme theories. Twenty-five health visitors, and twenty-two parents who had used the service, were recruited by a hospital administrator who had no direct role responsibilities to either group. All potential participants were sent a study information pack containing an invitation letter, information sheet, expression of interest form and a stamped self-addressed envelope. Interested participants were asked to complete and return the expression of interest form to the research team. Participants were then contacted directly to arrange a convenient date, time and venue for interviews. For logistical reasons, parents were offered an additional option to participate in a telephone interview. Informed consent was obtained from all participants. Interviews were audio recorded and all parents received £15 high street store vouchers. LD and SH conducted all interviews with health visitors and parents.

Interviews with participants centred on the initial programme theories and focused on identifying causal processes (mechanisms) and key elements of the contexts and how they reacted to produce outcomes. For instance, health visitors were asked their opinions of how the changes had influenced their practice and the perceived outcomes of the changes for children and families (see S2 Appendix. Topic guide for Health Visitors). Parents were asked about their perceptions and experiences of receiving the enhanced service and the effects on their families (see S3 Appendix. Topic guide for Parents).

Using the findings from phase two, the programme theories from phase one were disentangled, focusing on explaining ways in which the programme mechanisms unfolded or did not unfold in practice. This was presented as context, mechanism and outcome configurations to further aid understanding of the programme.

### Analysis

#### Phase 1

Within realist evaluation, analysis is driven by identifying causal processes (mechanisms) and key elements of the contexts and how they reacted to produce outcomes [13]. Using the focus group data we jointly produced a table and listed in the columns the key contexts, mechanisms and outcomes identified and formulated them as initial programme theories underpinning the EHV programme.

#### Phase 2

The analysis of health visitor and parents’ transcripts proceeded by seeking evidence to corroborate or refute the initial programme theories. A thematic analysis approach was used to analyse the interview data, using context, mechanism and outcome as imaging tool. Relevant extracts of transcripts from participants were independently coded by LD and SH through reading and re-reading each transcript. Where interpretation was difficult, consensus was reached through discussion with RJ. Similar codes were considered together and grouped under an overarching theme. The data within each theme were summarised and synthesised to produce understanding of how the EHV generated outcomes through the interaction of mechanisms and contexts. The analysis was facilitated by the software package NVivo 10 [23].

#### Phase 3 Refining the programme theory

This forms phase 3 of the realist design. We used the findings of phase 2 to revise the initial programme theories. Within realist evaluation, this phase is about explaining how the programme worked or did not work; and by clarifying where there were agreements and disagreements between the programme designers/implementers and the health visitors and parents who delivered and received it in practice. This section is outlined into five overarching components based on the findings from phase 2.

### Ethics approval and consent to participate

Ethics approval for the evaluation was granted by the University of Edinburgh Centre for Population Health Sciences Research Ethics committee and complied with research governance procedures in NHS Ayrshire and Arran.

## Results

### Initial programme theory

Three key programme theories that were identified, tested against our data and refined were:

1. By increasing the number of home visits by a single or small team of health visitors (context), families would develop trusting relationships with health visitors (mechanism), which can lead to greater awareness of needs (outcome) and support in a timely fashion (outcome).
2. Home visits (context) would offer health visitors a holistic perspective of the home environment (mechanism) and can facilitate early identification of concerns (outcome) and subsequent provision of tailored support (outcome).
3. The introduction of universal assessment pathways (context) offers systems and structures that standardise practice (mechanism) and ensure that the health visitors’ role is well defined and clear to families, wider agencies and themselves (outcome).

### Testing the programme theory

There were five main themes identified: 1) supporting families; 2) trusting relationships; 3) home visiting versus drop-in clinics; 4) role clarity; 5) systems and structures supporting the EHV. These themes focused on testing the three initial programme theories. The supporting families and trusting relationships themes covered initial programme theory one and the home visiting versus drop-in clinics theme was related to initial programme theory two. Further, initial programme theory three was covered by role clarity and systems and structures supporting the EHV themes.

#### Supporting families

Parents (P) highly appreciated the support provided by health visitors (HV). They felt that the increased, structured home visiting made it possible for them to receive more support both for themselves and their children.

I think it’s definitely improved since I’ve had my current child. This past year, I’ve had a lot of support because I went through postnatal depression with her and they (health visitors) were there supporting me as much as they could. In 2005 when I had my first one, I was just left. I mean, I had no support whatsoever (P16).

The programme ensured that families who required additional support, outwith the core visits, also received them. With the absence of drop-in clinics, parents found the option of contacting health visitors by phone quite positive and reassuring. Most parents mentioned that they have ultilised this opportunity.

Almost all the health visitors acknowledged that they found the structured, increased home visiting hugely beneficial in terms of the support it offered to children and families. They particularly appreciated the focus it gave them with regards to prevention and early identification of concerns.

I think it's made me far more aware of my families. I would have missed lots of things, and I would end up having lots of families in crisis. And it would be crisis intervention, rather than prevention, and that's not how to do it. A child should never get to the stage where their family is so chaotic that they have to be removed. So, for me, I wouldn't have been in the houses as often as I am now (HV23).

The majority of health visitors were keen to point out that with the previous service, concerns were often identified at advanced stages. Some health visitors added that the changes have placed them at a better position to build clear profiles of children and families’ concerns from the very beginning, where action could be taken if required, or possibly observe over time whether those concerns would be confirmed or allayed. Where concerns were confirmed, they believed they were able to provide targeted support or engage families with wider services through referrals.

#### Trusting relationships

There were conflicting opinions about whether the refocused role promoted trusting relationships in comparison with the previous role. Many of the health visitors however, believed that the home visits, integral to the enhanced service, ensured that families were more open and confident to discuss sensitive issues with them. This was substantiated by the view that families began to see them as their first point of contact on diverse health and wellbeing related issues.

Another mother asked me to go and see her and I saw her this morning. And the pretext was she wanted the baby weighed. But, really and truly, what she wanted to talk about was her eight year old child who has a bowel problem. So, I was able to discuss that, his diet, the importance of developing a bowel habit. So, the mother knows you well enough, trusts you enough, doesn’t think it’s important enough to go to the GP, so it saves an expensive doctor appointment if she feels she can contact me and I can manage the situation (HV17).

Some health visitors also added that, due to the trusting relationships they have developed, parents now feel more confident to seek more support through the phone.

I think we get to build up a better relationship with the parents. I think that's quite important, that we do sort of, at the beginning, we do six weekly visits, which I think is really helpful to build up that relationship. And you find mums do phone quite frequently now, and I think they've got a sort of a bit of a trust in you (HV18).

Parents supported this assertion. For example, one parent affirmed that the health visitor will be her first point of contact if she was concerned about any developmental or weight related issues as described below.

If it was concerns about development or weight or things, I would phone them first (P14).

The EHV is expected to promote continuity of care, with the same health visitor expected to fulfill the entire timeline, but in practice only a few parents mentioned that they had visits from the same health visitor. However, those who did, felt this promoted a better relationship with their health visitor.

I would say the relationship has been probably better for my current child because it's been continuously the same person whereas with first child it was just whoever did the clinic that morning (P14).

#### Home visiting versus drop-in clinics

Almost all the parents acknowledged that the home visits were hugely beneficial. They felt that health visitors gave them more attention, which was important in terms of discussing issues in more depth. This was also evident among parents who had previously experienced the drop-in clinics.

They don’t just rush in, like, let’s get the baby weighed. They don’t do that anymore, because they did that when I had my first one. It was, kind of like, in to do what they need to do and then go. Whereas now, it’s kind of, how are you doing? (P16).

It's quite personal I suppose but if they come into your house you can open up a bit more whereas if you come to the clinic and there's a big queue of people behind you waiting, you always think right I'm just gonna go in and get them weighed and go, but actually you want to discuss other things with them (P12).

However, a third of the parents who had previously experienced the drop-in clinics also stated that they missed attending them. The aspects of the drop-in clinics, which engendered such sentiments, included social networking opportunities and the possibility of weighing the baby on a more regular basis.

With the absence of drop-in clinics, some parents found the telephone support available outwith the home visiting schedule hugely beneficial.

…but even if I wanted to see her, I just need to phone her and say to her, listen, (name withheld), I’d like to see you. And she’d make an appointment and come out and see me, so it’s pretty much better (P19).

Health visitors felt that the benefits of the home visits were far greater than the previous drop-in clinics. A number of them acknowledged that the home visits put much more focus on families than the drop-in clinics, because they provide clear opportunities to identify concerns. They explained that it was impossible to observe and identify such concerns in the previous drop-in clinics.

Well, I think I had a family that I visited quite frequently at home, and the pattern became that this child was probably left alone quite a lot in the mornings. The parents weren't very good at getting out of their beds. And I think that became more apparent because I was visiting at home, because when you go at home, you hear the child crying, and the parents aren't responding. They took a long time to answer the door, and when you go in, the home environment is not great, the child is maybe running about with a really wet nappy on. Whereas, I think if it was a clinic setting, they might not come on time, however, the child will probably be well presented, because they know they're coming (HV18).

Some health visitors also felt that the home visits were hugely beneficial in terms of providing targeted support to children and their families.

One family I can think of off the top of my head, I’ve known that there’s been issues going on, but the fact that I’ve been going in more proactively, I’ve been able to get different services in and even getting those services in within two weeks. I’ve seen huge differences to that child. So whereas maybe previously, all that might not have been as timeously (HV22).

I used to think that we should still have kept our clinic going, but actually, see now, I'm happier doing the home visits. I don't mind doing the home visits, I think it gives you a better picture, because as I say, they could come to a clinic and be all completely nicely dressed, but you don't know what's going on in the background. So I do like the fact that all our visits are done at home (HV12).

Interestingly, even those who initially thought that the home visits were unnecessary and would not be beneficial to parents, as they felt it was too intrusive, acknowledged how valuable they have found it since it was implemented. Nevertheless, very few health visitors still believed that the drop-in clinics were more useful than the home visits.

#### Role clarity

Health visitors felt that the enhanced service has made their role much more explicit. They perceived that previously the role was unclear, but the changes have helped to clarify this.

I think health visiting, perhaps, arguably, before that was a profession that was maybe a bit harder to define, in very clear terms, exactly what your role was and where the boundaries between each of the areas were. So, I think it’s given us a lot more clarity in terms of our role and what we’re delivering and what we’re aiming to do (HV24).

More so, health visitors felt that families are now more knowledgeable of the range of services they offer.

And, I think, families are much more aware now of what to expect from the timeline so often they’ll phone us up and say, oh, my baby’s coming up for six months and, you know, they know that that’s what they’re entitled to now so, no, it’s good (HV16).

The role was however not only clearer to parents, but apparently improved professional partnership working too. Health visitors also mentioned that other agencies are now much clearer of their role.

So, if you are at say, for example, a child protection meeting and the team are drawing up a care plan, I think, we’re much clearer about what our role is within that whereas before we were probably getting, sort of, a lot of blurring of roles between the agencies and, kind of, getting fitted into the care plan whereas, I suppose, now we’re a bit clearer about what our role is (HV16).

#### Systems and structures supporting the EHV

There were a total of eleven visits within the enhanced service for core families. However, some health visitors were concerned that some of the gaps between home assessment visits in the timeline were too wide apart and that seemed uncomfortable for parents.

But a lot of the parents find from a year to 27 months is too long not to be seeing anybody. And I think that is quite a gap as well (HV12).

Almost all health visitors raised concerns about their current electronic recording system. They felt that a much more efficient and less laborious system would be helpful to compliment the arduous pathways.

I am constantly aware of the numbers of visits you’ve got in one day and it’s not just that, the electronic records take up quite a lot of time and, of course, it’s very important. You have not completed your intervention with a client until you’ve got your electronic record complete. You’ve got to have contemporaneous records (HV17).

There were small differences in service provision across areas of Ayrshire and Arran. There were also some indications that some areas were engaging the services of skill mix (support workers) and staff nurses to fulfil some of the pathways.

At the moment, we're using, sort of, skill mix to do some of the visits, some of the staff nurses are carrying out a couple of visits in the timeline (HV18).

A number of health visitors disapproved the use of skill mix and highlighted that involving skill mix was contrary to the concept of promoting continuity of care and consistency across the service.

I think it (timeline) has brought continuity I think, like, and within Ayrshire and Arran, you know, I think…I would hope that it would. It brings a more consistent approach to all families. However, talking to colleagues in other areas, I think some people have pulled back a bit more and have put in more skill mix and, you know, the waters are getting muddied again (HV13).

### Refined programme theory

#### Component 1: Supporting families

It was clear from both health visitors and parents that the increased, structured home visiting improved early identification of health and wellbeing concerns and assisted in terms of tailoring support to children and families (see Table 1). Health visitors were increasingly providing diverse kinds of support to children and families, including feeding and attachment support. Although most of the parents who participated in this study indicated that they did not breastfeed their children, they were nevertheless appreciative of the extensive feeding support they received from health visitors. This appears to be collaborated, although no cause and effect relationship is assumed, by recent figures, which depict that breastfeeding rates in NHS Ayrshire and Arran had increased since the enhanced service was implemented [24].

**Table 1:** Refined CMOs for supporting families.

Context	Mechanism	Outcome
Provision of structured universal pathway, with more focus on children aged 0–5 year	1. Families engaged more with HVs	1. Early identification of health and wellbeing concerns, resulted in the provision of tailored support, including feeding and attachment
	2. Additional support sought for families where necessary	2. More families engaging with wider services

#### Components 2: Trusting relationships

It was apparent that the enhanced service hugely improved trusting relationships, especially through the concept of continuity of care, where care is delivered by one or a small group of health visitors, for all or most planned episodes of care (see Table 2). Unlike drop-in clinics where different health visitors may run the clinics, home visits provided adequate time to establish rapport and develop strong relationships. This culminated in more parents recognising health visitors as first point of contact on a range of health and wellbeing issues in which they would have previously sought medical attention.

**Table 2:** Refined CMOs for trusting relationships.

Context	Mechanism	Outcome
Regular home visits by a single or a small team of HVs	Continuity of care ensured that barriers that prevented the discussion of sensitive issues were removed	Established trusted relationships increased confidence in HVs, with many recognised as first point of contact in diverse areas of concern

#### Component 3: Home visiting versus drop-in clinics

Parents were enthusiastic about the home visits, however a good number of those who had previously experienced the drop-in clinics indicated that they missed attending them because of the social support opportunities they provided. This was similar to the assertions made by health visitors. It appears that the removal of drop-in clinics limited parents’ accessibility to health visitors and in turn, revealed the wide gaps between some home assessment visits. The consequences of this was an increased demand for phone support as illustrated in Table 3.

**Table 3:** Refined CMOs for home visiting versus drop-in clinics.

Context	Mechanism	Outcome
Discontinuation of drop-in clinics and emergence of home visits	1. Holistic view of the home environment	1. Increased monitoring, prevention and identification of concerns
	2. Home visits promoted wide gaps between some home assessment visits	2. Limited accessibility to health visitors and increased demand for phone support

#### Components 4: Role clarity

There was evidence that the increased, structured home visiting service has clearly helped define the role of health visitors. Parents showed greater understanding of what they felt the health visitors’ role is. Health visitors also felt that they received more recognition from other professionals in terms of supporting the needs of children and families. They felt the programme raised their confidence and promoted a more efficient partnership working with other agencies, as depicted in Table 4.

**Table 4:** Refined CMOs for role clarity.

Context	Mechanism	Outcome
Enhanced service Streamlined and redefined the HV role	HV working along defined pathways made their role more clearer to both parents and other practitioners	Parents more confident of their expectations from the HV service; and HVs worked more efficiently with partner agencies

#### Component 5: Systems and structures supporting the EHV

The EHV standardised health visiting service in NHS Ayrshire and Arran. However, there appeared to be subtle differences in the service delivery across areas. There were also indications that some health visitors were referring more children and families to wider services as they struggled to accommodate the pathways and caseloads. Most health visitors found the referral pathways challenging and this contributed to the workload pressure, as outlined in Table 5.

**Table 5:** Refined CMOs for systems and structures supporting implementation.

Context	Mechanism	Outcome
Standardised operational guidance and procedure	1. HV assumed greater responsibilities in terms of delivering entire pathways	1. Perceived over referrals to other services due to challenges with workfoce capacity
	2. Consistency across the service but subtle differences existed due to workload pressure	2. Engaged skill mix and staff nurses to fulfil part of the pathways

## Discussion

This study was designed to evaluate how and why an EHV programme implemented in an early adopter site in Scotland could contribute to improving health and wellbeing outcomes for children and families. We used a realist evaluation approach, as it is considered a valuable alternative to the more traditional paradigm to understanding nursing and healthcare issues and provides an insight into how programmes work [25]. With health visiting practice playing a crucial role in health improvement and reducing health inequalities, this study identified some important dimensions of the EHV programme that can contribute to this agenda. The EHV appears to have improved early identification of health and wellbeing concerns amongst families who needed more support, leading to referral and engagement with sources of additional help. The home visiting context facilitated development of parent-health visitor relationships, as parents considered health visitors as their first point of contact on children’s wellbeing and developmental-related issues. More so, the EHV programme provided role clarity to health visitors, which in turn, enhanced collaborative partnerships. However, there were aspects of the EHV, which would require further consideration. For instance, both parents and health visitors were concerned about the wide gaps between some assessment visits and the cumbersome nature of the referral pathways.

Health visitors usually work in collaborative partnerships to develop local services and publicise them to families [26]. As such, health visitors are instrumental to uptake of services, especially for families who find services difficult to access [27]. It was clear in this evaluation that health visitors were making more referrals to wider services, including nursery placements, in ways, which were not previously possible. This approach emphasises the important role that health visitors can play in driving sustainable community development, by assisting individuals within communities to identify and mobilise existing, but often unrecognised assets and creating local opportunities [28, 29].

Collaborative partnerships also benefit where there are clarity of roles and clear responsibilities. This enables professionals to manage challenges around interagency or inter-professional team working [4]. In turn, families benefit from professionals working together in an effective way [30]. Health visitors felt that the enhanced service improved their professional partnership working. They perceived that other practitioners and services were also much clearer of their role.

The EHV places more emphasis on home visiting, rather than drop-in clinics, with all assessment visits expected in the client’s home. Cowley et al. [4] in their systematic review of health visiting practice suggested that aside from salutogenesis, a successful health visiting practice should also demonstrate a person-centred approach (human valuing) and recognise the person in the situation (human ecology). In order to achieve this, home visiting has an important role to play. In this evaluation, parents found the home visits beneficial, because it offered health visitors a unique opportunity to tailor care to their personal circumstances. Evidence also suggests that the home visits help to reduce anxiety amongst mothers regarding their child’s development and make them less reliant on health services [31, 32]. Although parents contacted health visitors by telephone for additional support, especially during long gaps between home visits, however recognising health visitors as their first point of contact has the potential to reduce pressure on health systems and expensive general practice visits.

Health visiting practice is dependent on parental participation, so the parent-health visitor relationship is a resource and an important part of health visiting practice [33]. The home environment is an ideal place for parents to develop relationships with practitioners, including health visitors [33, 34]. We found that home visiting ensured that families were more open and confident to discuss sensitive issues with health visitors. However, intertwined with client-professional relationship is the principle of continuity of care. Continuity of care is where the same person provides care for all, or most, planned episodes of care to promote trusting relationships between practitioners and clients [35]. Ideally, within the EHV programme, parents would receive all of their visits from the same health visitor. The few parents who had such experience, felt this helped to build better relationships with health visitors. However, many of the parents did not receive such service. In order to optimise the relationship formation opportunity inherent in home visiting, and to enhance the identification of emerging problems over time, more effort may be required to minimise the number of staff changes or handovers.

There have been recent debates in Scotland regarding the right balance between universal and targeted health visiting delivery. In 2005, policy changes reduced universal delivery and placed more emphasis on a targeted delivery model [36]. This reduced face-to-face visits from six to about three [37]. However, the Scottish Government later revised the guidance by introducing a further visit at 27–30 months [38]. Within this context, the EHV programme appears quite ambitious considering its eleven minimum visits for core families. Indeed, this has major implications for health visitors’ workload. Particularly, a study has suggested that health visitors’ workload influences their decision on how they identify children as requiring ‘additional’ support [37]. Reassuringly, the Scottish Government has committed to additional five hundred health visitors by 2018 [39].

Realist evaluation approach has gained traction in recent years, and has been successfully used to evaluate programmes in diverse fields, including health service research and others [40, 41]. However, the recent surge in the use of realist evaluation in nursing is a reflection of the valuable perspective this methodology provides into understanding the complexity within nursing practice and healthcare programmes [25]. Indeed, employing realist evaluation in this study has been useful in terms of unravelling attributes that are necessary to enhance health visiting practice.

This study was not designed to measure impact of the EHV and used only qualitative methods. As such, outcomes cannot be robustly linked to the activities offered by the programme, although it has identified factors, which could contribute to improving outcomes for children and families within health visiting practice. There has been very little research into users or parents’ experiences of health visiting service [42]. However, this evaluation used data from both parents and health visitors to provide useful insights into how the EHV service works.

Nearing the later stages of the evaluation, recruitment stalled and a member of staff at the study site contacted potential participants directly and informed them about the study. It is likely that this approach led to selection bias, in that individuals with particular characteristics might have agreed to participate in the study.

## Conclusion

Our study highlighted key benefits of the enhanced health visiting programme. The programme increased opportunities for monitoring and early identification of health and wellbeing concerns. It created structures for a more efficient partnership working and ensured that the needs of children and families were supported. These benefits need to be evaluated further in an effectiveness study.

## Supporting information

S1 Appendix Topic guide for stakeholders. (DOCX)

S2 Appendix Topic guide for health visitors. (DOCX)

S3 Appendix Topic guide for parents. (DOCX)

## Abbreviations

CMO: Context Mechanism Outcome
EHV: Enhanced Health Visiting
GIRFEC: Getting it Right For Every Child
HV: Health Visitor
NHS: National Health Service
