# Supplementary material for: Realist evaluation of an enhanced health visiting programme
Source: PLoS One. 2017 Jul 3;12(7):e0180569. doi: 10.1371/journal.pone.0180569 (PMC5495393; doi:10.1371/journal.pone.0180569)
Supplement: S1 Appendix — (DOCX) [file pone.0180569.s001.docx]

S1 Appendix. Topic guide for Stakeholders

| - What are the key changes that have been introduced to Health Visiting since April 2013? - What are the key contents that the service will provide over and above what was previously offered? - What do you think was the rationale for implementing these new changes? - What additional resources were needed to implement these changes? - How was it envisaged that the changes would make things better for: - Health visiting practice? - Children and families? - How do you think delivery of the revised universal pathway home visits by Health Visitors could improve outcomes for children and families? - How are the changes going to contribute in terms of keeping children safe and also promote their wellbeing? - What specific plans/structures have been put in place to ensure that Health Visitors improve their knowledge and awareness of community assets, care and referral pathways? - What strategies or activities were put in place before the changes were introduced? - Could you tell me how these changes were implemented? - Were there any partners involved in planning or implementing these changes? If so, could you tell me how they were involved? - What do you think are the challenges to implementing and delivering this enhanced service? - What organisational structures do you think might act as barrier to these enhanced health visiting service?   - How are you addressing them?   - What do you think have been the key benefits of implementing these changes to the health visiting service in NHS Ayrshire and Arran? - What were you expecting to achieve in the short, medium and long term? |
| --- |
