# Supplementary material for: Realist evaluation of an enhanced health visiting programme
Source: PLoS One. 2017 Jul 3;12(7):e0180569. doi: 10.1371/journal.pone.0180569 (PMC5495393; doi:10.1371/journal.pone.0180569)
Supplement: S2 Appendix — (DOCX) [file pone.0180569.s002.docx]

S2 Appendix. Topic guide for Health Visitors

| 1. How is your service responding to the national drive for early intervention and prevention, to ensure that all children have the best start in life? 2. I understand that you are now delivering a structured, increased home visiting service for all families, apart from workload issues what difference has this made for: 3. Children 4. The families 5. Professional partnership working 6. You as the Health Visitor   3. What opportunities does a home visiting programme provide you with? Are there specific interventions that you are able to deliver? (prompt: in terms of assessing the child’s needs and risks?)  4. Neuro-development, child development and attachment are of key importance to give children the best start in life.   1. How has your individual practice changed in response? 2. How has service delivery changed in response? 3. Do you have any other suggestions to enhance professional learning around Neuro-development, child development and attachment?   5. Has the service you provide enabled you to link to wider services such as nurseries and social work in ways that you had not previously done?  6. Can you give me some examples of interventions or supports that you have been able to access earlier for children and families due to the more frequent contacts?  7. Does the home visiting service and universal contacts allow you to:   1. Strengthen relationships with families? 2. Improve outcomes for children and families? 3. If so how?   8. Has the increased visit schedule affected other parts of your work? If so, in what ways?  9. We know that the introduction of an enhanced home visiting programme in Ayrshire has been challenging due to workload and high caseloads. However when Ayrshire has the additional 50 Health Visitors what will you be able to provide that you are currently unable to do, due to workload constraints?  10. What are the differences between assessing children in their home to a clinic setting? |
| --- |
