# Supplementary material for: Realist evaluation of an enhanced health visiting programme
Source: PLoS One. 2017 Jul 3;12(7):e0180569. doi: 10.1371/journal.pone.0180569 (PMC5495393; doi:10.1371/journal.pone.0180569)
Supplement: S3 Appendix — (DOCX) [file pone.0180569.s003.docx]

S3 Appendix. Topic guide for Parents

| 1. What age is your child/children? 2. How do you feel about the number of visits you have received from your HV so far? 3. Have these visits been carried out by the same Health Visitor? If not do you know why? 4. If you have other children, has your experience of having a Health Visitor changed? 5. How do you feel about the quality of visits you have received? Do you feel supported by your HV? 6. Can you tell me what support you received from your Health Visitor? Did the HV help you with issues/concerns that were important to you? 7. What support did you receive from your Health Visitor regarding feeding your child?    1. Was this enough?    2. If not, what else would have helped? 8. Do you feel that your views are always considered as part of your child’s assessment? 9. Are you able to contact your Health Visitor with any concerns? 10. Has your Health Visitor helped in terms of engaging your family with other services or professionals? If so how? 11. What is the most important part of having a Health Visitor to you? |
| --- |
